# Progressive resistance of BTK-143 osteosarcoma cells to Apo2L/TRAIL-induced apoptosis is mediated by acquisition of DcR2/TRAIL-R4 expression: resensitisation with chemotherapy

**DOI:** 10.1038/sj.bjc.6601021

**Published:** 2003-07-01

**Authors:** S Bouralexis, D M Findlay, G J Atkins, A Labrinidis, S Hay, A Evdokiou

**Affiliations:** 1 Department of Orthopaedics and Trauma, University of Adelaide, Level 4, Bice Building, Royal Adelaide Hospital, North Terrace, Adelaide 5000, South Australia, Australia

**Keywords:** DcR2, Apo2L/TRAIL, osteosarcoma, resistance, chemotherapy, apoptosis

## Abstract

Apo2 ligand (Apo2L, also known as TRAIL) is a member of the tumour necrosis factor (TNF) family of cytokines that selectively induces the death of cancer cells, but not of normal cells. We observed that recombinant Apo2L/TRAIL was proapoptotic in early-passage BTK-143 osteogenic sarcoma cells, inducing 80% cell death during a 24 h treatment period. Apo2L/TRAIL-induced apoptosis was blocked by caspase inhibition. With increasing passage in culture, BTK-143 cells became progressively resistant to the apoptotic effects of Apo2L/TRAIL. RNA and flow cytometric analysis demonstrated that resistance to Apo2L/TRAIL was paralleled by progressive acquisition of the decoy receptor, DcR2. Blocking of DcR2 function with a specific anti-DcR2 antibody restored sensitivity to Apo2L/TRAIL in a dose-dependent manner. Importantly, treatment of resistant cells with the chemotherapeutic agents doxorubicin, cisplatin and etoposide reversed the resistance to Apo2L/TRAIL, which was associated with drug-induced upregulation of mRNA encoding the death receptors DR4 and DR5. BTK-143 cells thus represent a useful model system to investigate both the mechanisms of acquisition of resistance of tumour cells to Apo2L/TRAIL and the use of conventional drugs and novel agents to overcome resistance to Apo2L/TRAIL.

Osteosarcoma is the most common primary malignancy of the skeleton. The first choice of treatment for osteosarcoma is preoperative chemotherapy (Seki *et al*, 2000). The agents doxorubicin (DOX), cisplatin (CDDP) and etoposide (ETP), and others such as cyclophosphamide, methotrexate and vincristine, are commonly used in combination therapy to target osteosarcoma (Bramwell, 2000; Seki *et al*, 2000). However, the use of chemotherapy is often associated with the frequent acquisition of drug-resistant phenotypes and the occurrence of ‘second malignancies’. In addition, the associated cytotoxic side effects on normal tissues and organs represent a serious limitation to the use of these agents. Importantly, osteosarcomas are often resistant to the induction of apoptosis by anticancer agents (Seki *et al*, 2000), so that there is a pressing need to develop new and alternative approaches to the current chemical treatment of this tumour type.

Apo2L/TRAIL is a new member of the tumour necrosis factor (TNF)-cytokine family that induces cell death in a wide variety of tumour cell lines, but does not seem to be cytotoxic to many normal cell types *in vitro* or *in vivo* (Ashkenazi *et al*, 1999; Walczak *et al*, 1999; Atkins *et al*, 2002; Evdokiou *et al*, 2002). Apo2L/TRAIL is a type II transmembrane protein that induces apoptosis through interactions with its death domain-containing receptors, DR4/TRAIL-R1 and DR5/TRAIL-R2 (Pan *et al*, 1997a,1997b; Sheridan *et al*, 1997; Walczak *et al*, 1997). Ligand-dependent activation of DR4 and DR5 involves receptor multimerisation, with subsequent recruitment of the intracellular death adaptor molecule, Fas-associated death domain protein (FADD), that engages the initiator protease caspase-8 (Newton *et al*, 2001). Fas-associated death domain protein triggers caspase-8 autoactivation and subsequently leads to activation of downstream effector caspases, including caspase-3 and caspase-7, resulting in the cleavage of cellular substrates and ultimately cell death by apoptosis (Nagata, 1997; Ashkenazi and Dixit, 1998). Death receptor activity can be antagonised by the so-called ‘decoy’ receptors for Apo2L/TRAIL, of which there are to date three known in the human: DcR1/TRAIL-R3/TRID (Degli-Esposti *et al*, 1997a,1997b), DcR2/TRAIL-R4/TRUNDD (Marsters *et al*, 1997; Pan *et al*, 1998; Degli-Esposti, 1999) and osteoprotegerin (OPG) (Emery *et al*, 1998). DcR1 and DcR2 lack functional death domains and cannot mediate apoptosis. Osteoprotegerin is a widely expressed soluble member of the TNF receptor family that is capable of binding to Apo2L/TRAIL and can block Apo2L/TRAIL-induced apoptosis (Emery *et al*, 1998), although its role in doing so has not been extensively explored.

The relative levels of expression of death and decoy receptors suggests a possible mechanism, by which cells are rendered resistant to Apo2L/TRAIL, although the pattern of expression of Apo2L/TRAIL receptors does not necessarily correlate with resistance or sensitivity to Apo2L/TRAIL-mediated apoptosis (Degli-Esposti, 1999; Keane *et al*, 1999). Recent findings, which demonstrate that the death and decoy receptors for Apo2L/TRAIL have different subcellular locations and undergo redistribution within the cell following treatment with Apo2L/TRAIL (Zhang *et al*, 2000a,2000b), highlight other levels of complexity that determine Apo2L/TRAIL sensitivity. These, and other observations suggest that, in addition to Apo2L/TRAIL receptor expression and distribution, the cytotoxic effects of Apo2L/TRAIL are likely to be mediated by events involving the complex interplay between proapoptotic and prosurvival pathways (Roy and Nicholson, 2000).

Irrespective of the mechanisms that determine sensitivity or resistance, animal studies indicate that Apo2L/TRAIL has great therapeutic potential for many cancer types (Ashkenazi *et al*, 1999). Apo2L/TRAIL was demonstrated safe and nonimmunogenic (Ashkenazi *et al*, 1999; Walczak *et al*, 1999), was active alone in some cancer types (Ashkenazi *et al*, 1999) and exhibited synergistic activity with chemotherapeutic agents, causing marked regression or complete remission of tumours (Ashkenazi *et al*, 1999; Walczak *et al*, 1999). However, some tumour types are resistant to Apo2L/TRAIL-induced apoptosis (Bin *et al*, 2002), while it has been reported that melanoma cells are frequently resistant at the time of surgical excision, but regain sensitivity in cell culture (Nguyen *et al*, 2001). It is therefore important to develop an understanding of the factors that lead to sensitivity or resistance of tumour cells to Apo2L/TRAIL.

Using a panel of human osteosarcoma cell lines, we recently observed resistance of this type of tumour cell to Apo2L/TRAIL, which could be overcome by cotreatment with chemotherapeutic agents (Evdokiou *et al*, 2002). However, we observed initially that recombinant Apo2L/TRAIL was proapoptotic in one cell line, the BTK-143 osteosarcoma cell line. The present study found that with repeated passage in culture, BTK-143 cells became progressively resistant to the apoptotic effects of Apo2L/TRAIL. Further analysis revealed that there was a significant increase in the expression of DcR2 with repeated passage, which paralleled with a loss of sensitivity to Apo2L/TRAIL, and suggested a role for DcR2 in determining resistance to Apo2L/TRAIL-induced apoptosis. Importantly, treatment of the late passage resistant cells with DOX, CDDP or ETP reversed the resistance to Apo2L/TRAIL and sensitised late passage cells to Apo2L/TRAIL-induced apoptosis. These cells therefore represent a useful model system to investigate the mechanisms of acquisition of resistance of tumour cells to Apo2L/TRAIL and those by which resistance may be overcome.

## MATERIALS AND METHODS

### Cells and reagents

The BTK-143 osteogenic sarcoma cell line was obtained from the American Type Culture Collection (ATCC, Rockville, MD, USA) and cultured in Dulbecco's modified Eagle's medium (DMEM), supplemented with glutamine (2 mM), penicillin (100 IU ml^−1^), streptomycin (100 *μ*g ml^−1^), gentamicin (160  *μ*g ml^−1^) and 10% foetal bovine serum (Biosciences, Sydney, Australia). Cultures were grown in a humidified atmosphere containing 5% CO_2_. The cells were passaged every 3–4 days, after reaching 80% confluency, into T 75 cm^2^ tissue culture flasks (Corning, Costar Corp., Cambridge, MA, USA). Nontagged homotrimeric Apo2L/TRAIL was generously provided by Genentech, Inc. (South San Francisco, CA, USA). Doxorubicin, CDDP, and ETP were obtained from Pharmacia & Upjohn (Kalamazoo, MI, USA). The tetrapeptide caspase inhibitors ZVAD-fmk and ZDEVD-fmk were purchased from Calbiochem (Alexandria, NSW, Australia).

### Measurement of cell number

To determine effects on cell number, 2.5 × 10^4^ cells per well were seeded in 48-well microtitre plates and allowed to adhere to the plate overnight. Cells were then treated for 24 h with 100 ng ml^−1^ of soluble recombinant Apo2L/TRAIL. Cell number was determined by staining the cells with crystal violet and measuring OD_570 nm_ of cell lysates. To assess the effects of chemotherapeutic agents on Apo2L/TRAIL-mediated effects on cell number, cells were plated in 48-well plates and allowed to adhere for 24–48 h. Doxorubicin, CDDP or ETP were added to the wells at the indicated concentrations either alone, or in combination with 100 ng ml^−1^ of Apo2L/TRAIL, and incubated for 24 h. Cell number was again determined by staining the cells with crystal violet. All cell number experiments were performed in triplicate or quadruplicate and experiments were repeated at least three times. Results of representative experiments are given as the mean ±s.d.

### Reverse transcription–polymerase chain reaction analysis

RNA was extracted from cells using the Trizol Reagent (Invitrogen, Groningen, Netherlands), as recommended by the supplier. First-strand complementary DNA (cDNA) was synthesised from 2.0 *μ*g of total RNA in a final volume of 20 *μ*l using SuperScript II (Life Invitrogen, BV CH, Groningen, the Netherlands) and random primers (Bresagen Inc., Adelaide, South Australia). cDNA (1.0 *μ*l out of 20 *μ*l) was then amplified by polymerase chain reaction (PCR) using specific primers corresponding to mRNA encoding human gene products as outlined in Table 1

**Table 1:** PCR primers and conditions for the specific amplification by RT–PCR of human mRNA species

*Target gene*	*Primer sequences sense (S), antisense (AS)*	*Annealing temperature (°C)*	*Product size (kb)*
DR4	S 5′-TGCTGCAGCTCGTACCTAGCTC-3′	66	646
	AS 5′-TTGCTGCTCAGAGACGAAAGTGG-3′		
DR5	S 5′-CTGCAACTGTGACTCCTATG-3'	66	424
	AS 5'-GTCTGCTCTGATCACCCAAC-3′		
DcR1	S 5′-TCCTAGCTTACTCTGCCACCACT-3′	66	700
	AS 5′-CACAATTAGAACTATGATCCCTACG-3′		
DcR2	S 5′-TTCTCATGGGACTTTGGGGACAA-3′	66	855
	AS 5′-CTGTTACTCAGGGTCTCGTTGC-3′		
OPG	S 5′-TGCTGTTCCTACAAAGTTTAC-3′	62	435
	AS 5′-CTTTGAGTGCTTTAGTGCGTG-3′		
FLIP	S 5′-AATTCAAGGCTCAGAAGCGA-3′	62	229
	AS 5′-GGCAGAAACTCTGCTGTTCC-3′		
GAPDH	S 5′-CATGGAGAAGGCTGGGGCTC-3′	62	414
	AS 5′-CACTGACACGTTGGCAGTGG-3′		

S=sense primers; AS=antisense primers. Annealing temperatures were determined empirically using AmpliTaq Gold (Perkin Elmer) in a gradient thermal cycler (Corbett Research).

. The 20 *μ*l amplification mixture contained 1 U of Ampli*Taq* Gold DNA polymerase (Perkin Elmer, Norwalk, CT, USA), 100 ng each of the 5′ and 3′ primers, 0.2 mM dNTPs (Pharmacia Biotech, Uppsala, Sweden), 1.5 mM MgCl_2_, 2 *μ*l 10 × reaction buffer and sterile DEPC-H_2_O. Polymerase chain reaction was performed for 23 cycles for GAPDH and 30–35 cycles for other primer pairs, such that all products could be assayed in the exponential phase of the amplification curve, in a thermal cycler (Corbett Research, Melbourne, Victoria, Australia). After an initial step at 95°C for 9 min to activate the polymerase, each cycle consisted of 1 min of denaturation at 94°C, 1 min of annealing at the temperatures indicated in Table 1, and 1 min of extension at 72°C. This was followed by an additional extension step at 72°C for 1 min. Primer sequences and predicted PCR product sizes are shown in Table 1. Amplification products were resolved by electrophoresis on a 2% w v^−1^ agarose gel and poststained with SYBR-1 Gold (Molecular Probes, Eugene, OR, USA). The relative amounts of the PCR products were determined by quantifying the intensity of bands using a FluorImager and ImageQuant software (Molecular Dynamics, Sunnyvale, CA, USA). Amplified products are represented as a ratio of the respective PCR product/GAPDH PCR product. To show that there were no false-positive results, PCR reactions were carried out using nonreverse transcribed RNA, and on reaction mixtures to which no RNA was added.

### Measurement of DEVD-caspase like activity

DEVD-caspase-3 like activity was assayed by cleavage of zDEVD-AFC (z-asp-glu-val-asp-7-amino-4-trifluoro-methyl-coumarin), a fluorogenic substrate based on the peptide sequence at the caspase-3 cleavage site of poly (ADP-ribose) polymerase (Medina *et al*, 1997). Cells (2.5 × 10^4^) grown in 48-well plates were treated as indicated, washed once with HBSS and resuspended in 200 *μ*l of NP-40 lysis buffer containing 5 mM Tris-HCl, 5 mM EDTA and 0.5% NP-40, pH 7.5. After 15 min in lysis buffer at 4°C, insoluble material was pelleted at 15 000 **g** and an aliquot of the lysate was tested for protease activity. To each assay tube containing 8 *μ*M of substrate in 1 ml of protease buffer (50 mM HEPES, 10% sucrose, 10 mM DTT, 0.1% CHAPS, pH 7.4), was added to 20 *μ*l of cell lysate. Reactions were allowed to proceed for 4 h at room temperature in total darkness, whereupon fluorescence was quantified (Exc 400, Emis 505) in a Perkin-Elmer LS50 spectrofluorimeter. Optimal amounts of added lysate and duration of assay were taken from linear portions of curves as determined in preliminary experiments. One unit of caspase activity was taken as one fluorescence unit (at slit widths of 12.5 nm) per 4 h incubation with substrate.

The tetrapeptide caspase inhibitors ZVAD-fmk, and ZDEVD-fmk were resuspended in DMSO (Sigma-Aldrich, Castle Hill, NSW, Australia) and were added to cells at the indicated concentrations 30 min before treatment with Apo2L/TRAIL. Control cells were incubated with the equivalent concentration of DMSO.

### 4′,6-diamidine-2′-phenylindole dihydrochloride staining of nuclei

Cells were seeded on plastic chamber slides and treated as indicated. After two washes with phosphate-buffered saline (PBS), cells were fixed in an ethanol/acetic acid solution (six parts ethanol : one part acetic acid) for 10 min, washed again with PBS, and allowed to air-dry overnight. The chamber slides were then incubated with 0.8 *μ*g ml^−1^ of 4′,6-diamidine-2′–phenylindole dihydrochloride (DAPI), (Roche Diagnostics, Castle Hill, NSW, Australia) in PBS for 2-5 min in the dark and at room temperature. After several washes in PBS, the coverslips were mounted with *n*-propyl gallate (antifade, Sigma Chemical Company, St Louis, MO, USA). 4′,6-diamidine-2′-phenylindole dihydrochloride staining was visualised by fluorescence microscopy.

### Flow cytometric analysis

For flow cytometric analysis, cells were seeded into fresh culture flasks 1 day prior to the assay, rinsed twice with PBS and detached using 2 mM EDTA in PBS at 37°C for 5 min. All subsequent incubation steps were performed on ice and centrifugation steps were performed at 4°C. Cells were washed twice in PBS by centrifugation at 200 **g** for 5 min, resuspended at 2 × 10^6^ cell ml^−1^ in blocking buffer (10% BSA/PBS + 0.1% azide) and 50 *μ*l aliquots of the cell suspensions were added to polypropylene FACS tubes. To each of these was added 50 *μ*l of monoclonal antibody solution (Mab) specific for human TRAIL receptors TR1, TR2, TR3, TR4 (Zhang *et al*, 2000a,2000b) (supplied by Immunex Corp., Seattle, WA, USA) or isotype-matched nonbinding control Mabs (provided by Dr Leonie Ashman, University of Newcastle, NSW, Australia), each diluted to 10 *μ*g ml^−1^ in blocking buffer. After incubation for 45 min, the cells were washed twice in wash buffer by centrifugation at 300 **g**. To the resuspended cell pellets was added 50 *μ*l of FITC-labelled F(ab′)_2_ sheep anti-mouse Ig or goat anti-mouse IgG-PE (Southern Biotechnology, Birmingham, AL, USA), both diluted 1 : 50 in blocking buffer. The cells were incubated for a further 45 min in the dark, washed twice as above, then resuspended and fixed in 0.5 ml of cold 1% w v^−1^ paraformaldehyde for analysis by flow cytometry.

### *In situ* immunofluorescence

Cells were seeded into 8-chamber slides (Nunc, Inc., Naperville, IL, USA) at 1 × 10^4^ cells per well, and cultured for 24 h. *In situ* immunofluorescence was performed at room temperature. Cells were rinsed once in PBS, fixed in 2% paraformaldehyde in PBS for 5 min, and permeabilised with 0.1% saponin in PBS containing 10% heat-inactivated pooled normal human serum (permeabilisation buffer), for 10 min. The cells were then washed thrice with PBS containing 0.1% bovine serum albumin (BSA) and 0.1% NaN_3_ (wash buffer), and blocked in 5% normal goat serum (NGS) containing 0.1% w v^−1^ NaN_3_ (blocking buffer) for 60 min. The blocking buffer was removed and monoclonal antibodies (Mab) specific for human TRAIL receptors TR1, TR2, TR3, TR4 (as above), OPG (Mab 8051 or isotype-matched nonbinding control Mabs (as above), each diluted to 10 *μ*g ml^−1^ in blocking buffer as above, were added for 60 min. The slides were washed thrice in PBS containing 0.05% (v v^−1^) Triton X-100. To reveal primary antibody reactivity, cells were incubated for a further 60 min in the dark, with a 1 : 30 dilution in PBS of FITC-conjugated goat anti-mouse F(ab′)_2_. The cells were washed thrice as above, then resuspended and fixed in 0.2 ml of cold 1% w v^−1^ paraformaldehyde for analysis. The labelled samples were mounted in Univert mountant and examined using an Olympus Bx 51 fluorescence microscope and imaged using a Photometrics Coolsnap Fx digital camera (Roper Scientific, NJ, USA).

### Inhibition of function of DcR2

Cells were seeded at 2.5 × 10^4^ cells per well in 48-well microtitre plates and allowed to adhere overnight. The cells were then cultured in DMEM (as above) with increasing concentrations of anti-DcR2 antibody or an isotype matched negative control antibody (as above), of up to 100 *μ*g ml^−1^ for a period of 24 h. Culture media were then removed and the cells treated for a following 24 h with 100 ng ml^−1^ of recombinant soluble Apo2L/TRAIL.

Cell numbers were determined by staining the cells with crystal violet and measuring OD_570 nm_ of cell lysates, as we have described previously (Evdokiou *et al*, 2002). In some experiments, relative cell numbers were also ascertained by staining with WST-1 (Roche Diagnostics, Mannheim, Germany), which gave identical results to those obtained with crystal violet (data not shown). All cell proliferation experiments were performed in triplicate and experiments were repeated at least three times. Results of representative experiments are given as the mean ±s.d.

## RESULTS

### Sensitivity of BTK-143 osteosarcoma cells to recombinant soluble Apo2L/TRAIL

**Figure 1:**
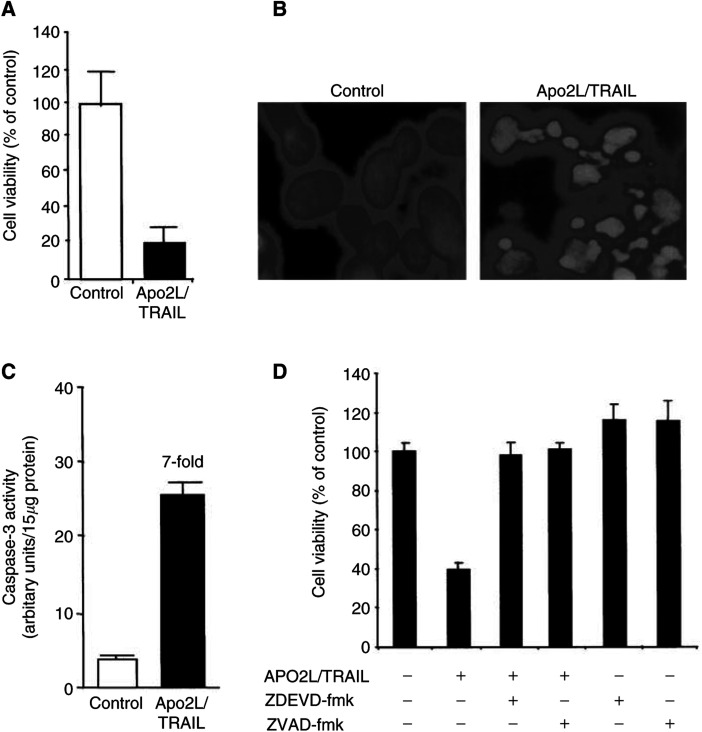
Apo2L/TRAIL-induced apoptosis in BTK-143 cells. (**A**) BTK-143 osteosarcoma cells (1.5 × 10^4^) were seeded into triplicate wells of a 48-well plate. Cells were incubated for 24 h in the absence (white bar) or presence of 100 ng ml^−1^ of soluble recombinant Apo2L/TRAIL (black bar). Cell number was assessed by crystal violet staining and the results are given as a percentage of control, untreated cells. Data shown are means of triplicate wells ±s.d. and are representative of experiments repeated at least three times. (**B**) DAPI nuclear fluorescence stain, showing changes in the nuclei of Apo2L/TRAIL-treated BTK-143 cells consistent with induction of apoptosis. Left panel: Control, untreated cells showing homogeneously fluorescent nuclei. Right panel: Cells treated for 24 h with 100 ng ml^−1^ Apo2L/TRAIL; note the presence of apoptotic bodies containing condensed chromatin. (**C**) Caspase-3-like activity in BTK-143 cells incubated in the absence (white bar) or treated for 24 h with 100 ng ml^−1^ of Apo2L/TRAIL (black bar). Activity was determined in cell lysates using the caspase-3-specific fluorogenic substrate, zDEVD-AFC, as described in the Methods. Data shown are representative of three independent experiments: bars, ±s.d. (**D**) Effect of caspase inhibitors on Apo2L/TRAIL-treated BTK-143 cells. Cells were treated for 24 h with 100 ng ml^−1^ of Apo2L/TRAIL alone or with Apo2L/TRAIL and either the pan-caspase inhibitor zVAD-fmk (50 *μ*M) or the caspase-3 specific inhibitor zDEVD-fmk (50 *μ*M). Cells were also treated with each inhibitor alone. Cell viability is expressed as a percentage of control untreated cells. Data are means of triplicate results from a representative experiment repeated at least three times; bars, ±s.d.

Treatment of BTK-143 cells with recombinant Apo2L/TRAIL, at a concentration of 100 ng ml^−1^ for 24 h, resulted in a considerable reduction in cell number, with 80% cell death compared with untreated control cells (Figure 1A

). Morphological changes characteristic of apoptosis, including chromatin condensation, nuclear fragmentation and the formation of dense rounded apoptotic bodies, were evident in a high percentage of the Apo2L/TRAIL-treated cells following DAPI staining (Figure 1B). The onset of apoptosis following treatment with Apo2L/TRAIL was concomitant with a seven-fold increase in the level of caspase-3-like activity (Figure 1C). The pan-caspase inhibitor, zVAD-fmk, and the caspase-3-specific inhibitor, zDEVD-fmk completely prevented the Apo2L/TRAIL-induced apoptosis confirming the role of caspase activation in Apo2L/TRAIL-mediated apoptosis of BTK-143 cells (Figure 1D).

**Figure 2:**
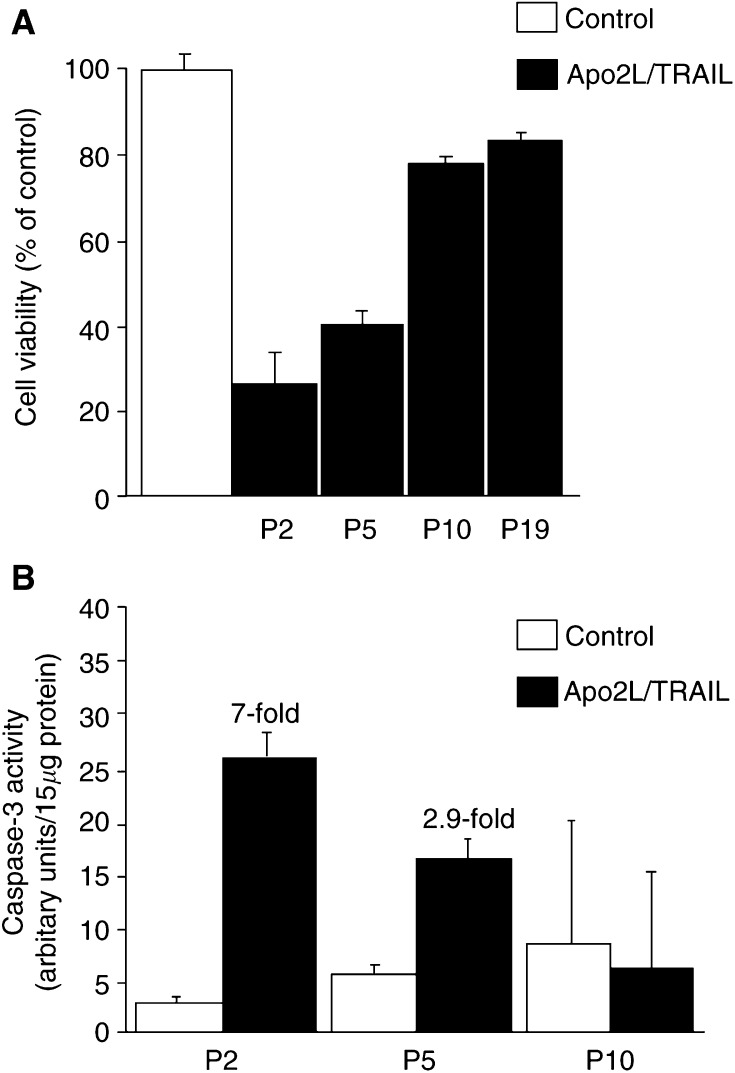
Repeated passage of BTK-143 cells leads to resistance to Apo2L/TRAIL-induced apoptosis and loss of caspase activation. Apo2L/TRAIL-sensitive BTK-143 cells were treated for 24 h with 100 ng ml^−1^ of Apo2L/TRAIL. (**A**) Cell number, represented as a function of total protein stained with crystal violet, is shown as a percentage of control untreated cells for the passage (P) numbers indicated. (**B**) Caspase-3-like activity in cell lysates prepared from control or Apo2L/TRAIL-treated cells, as indicated. Caspase-3-like activity was determined using the caspase-3 specific fluorogenic substrate, zDEVD-AFC, as described in the Materials and Methods. Results represent caspase activation per 15 *μ*g lysate protein. Data are means of triplicate results from a representative experiment repeated at least three times; bars, ±s.d.

With repeated passage in culture, we found that BTK-143 cells became progressively resistant to the apoptotic effects of Apo2L/TRAIL. For example, at passage 2, treatment of these cells for 24 h resulted in 80% cell deaths (Figure 2A

). By passage 5, only 60% of the cells were responsive and at passage 10 only 20% of the cells were responsive to Apo2L/TRAIL, which remained the case at least up to passage 19 (Figure 2A). Assessment of caspase-3-like protease activity demonstrated a gradual decrease in the level of caspase-3 activation by Apo2L/TRAIL as the passage number increased (Figure 2B).

### Apo2L/TRAIL receptor mRNA expression and Apo2L/TRAIL sensitivity

**Figure 3:**
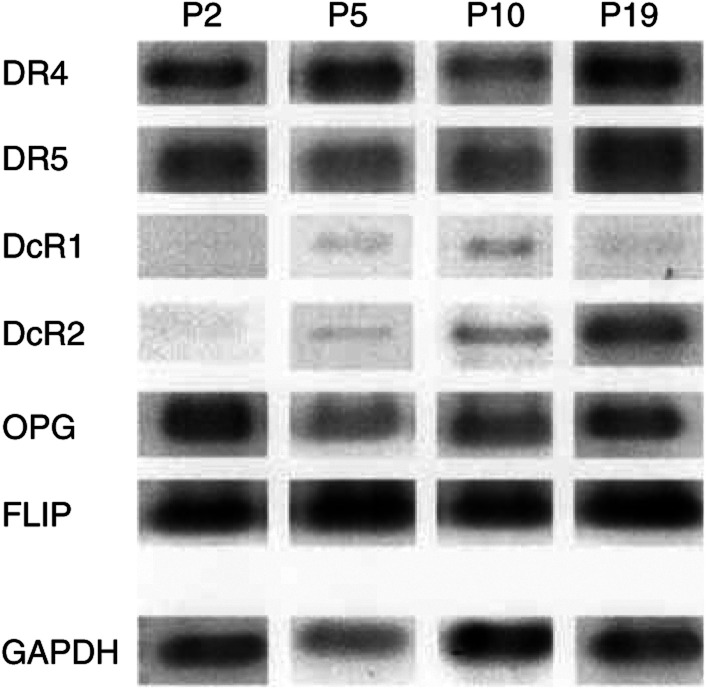
Reverse transcriptase–polymerase chain reaction analysis of Apo2L/TRAIL receptor mRNA expression patterns in BTK-143 osteosarcoma cell line with increasing passage. Analysis of steady-state Apo2L/TRAIL receptor mRNA expression in the BTK-143 osteosarcoma cells, assessed by semiquantitative RT–PCR analysis, as described in the Materials and methods. Expression of the housekeeping gene, GAPDH, served to normalise for starting mRNA. The panel shows the pattern of Apo2L/TRAIL receptor expression with increase in passage (P) number. Note the strong expression of DcR2 at P19.

To determine whether there were any changes in Apo2L/TRAIL receptor expression with passage number in BTK-143 cells, semiquantitative reverse transcriptase–polymerase chain reaction (RT–PCR) analysis was performed to amplify mRNA corresponding to each of the Apo2L/TRAIL receptors. Preliminary experiments were performed to ensure that the number of PCR cycles used in each case was within the linear phase of each amplification curve (data not shown). Reverse transcriptase–polymerase chain reaction analysis of BTK-143 cells at passage 2, 5, 10 and 19 revealed that the mRNA level of the death receptors, DR4 and DR5, did not notably change with passage (Figure 3

). However, with increasing passage number, the level of mRNA corresponding to decoy receptors, DcR1 and DcR2, increased markedly, with the effect on DcR2 expression being more pronounced (Figure 3). At passage 2, there was little or no detectable DcR1 mRNA and this increased to readily detectable levels by passage 10. More dramatically, the level of DcR2 mRNA progressively increased from undetectable levels at early passage to high levels at later passage (Figure 3). It has been suggested that sensitivity to Apo2L/TRAIL might be explained by downstream components of the Apo2L/TRAIL apoptotic pathway. We therefore examined whether there was any change in the steady-state level of FLIP mRNA (a known inhibitor of the Apo2L/TRAIL apoptotic pathway), between early-passage (sensitive) and late-passage (resistant) cells. Reverse transcriptase–polymerase chain reaction analysis demonstrated that the BTK-143 cells expressed high levels of FLIP mRNA, which did not significantly change with increasing passage number (Figure 3).

**Figure 4:**
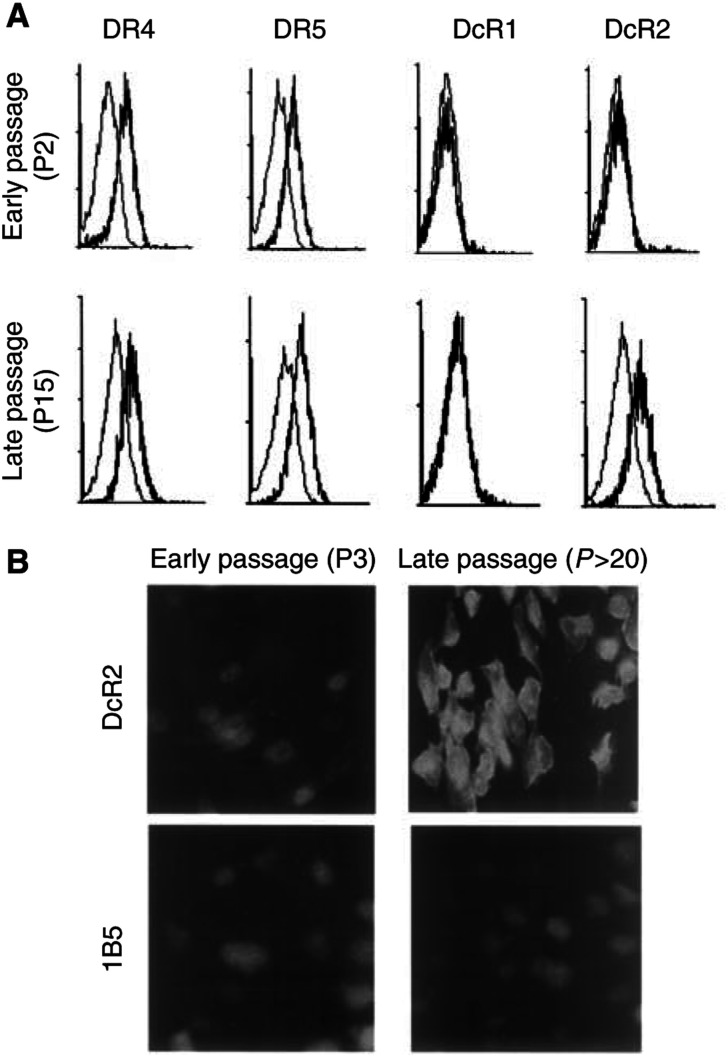
Cell surface expression of Apo2L/TRAIL receptors detected by flow cytometric analysis and *in situ* immunofluorescence staining. (**A**) Flow cytometric data of cell surface expression of Apo2L/TRAIL receptors shows strong expression of DcR2 by late-passage (p15) cells, compared with early-passage (P2) cells (heavy line). The 1B5 isotype-matched (IgG1) murine monoclonal antibody was used as a negative control (light line). These results are from a representative experiment repeated at least three times. (**B**) *In situ* immunofluorescence of DcR2 in early-(P3) and late-passage (P>20) BTK-143 cells. Note the strong expression of DcR2 at the cell surface and within the cytoplasm of the late-passage cells, compared with the weak or absent staining of early-passage (P3) cells. The 1B5 isotype-matched (IgG1) murine monoclonal antibody shows only weak staining that is no different between early- and late-passaged cells.

**Figure 5:**
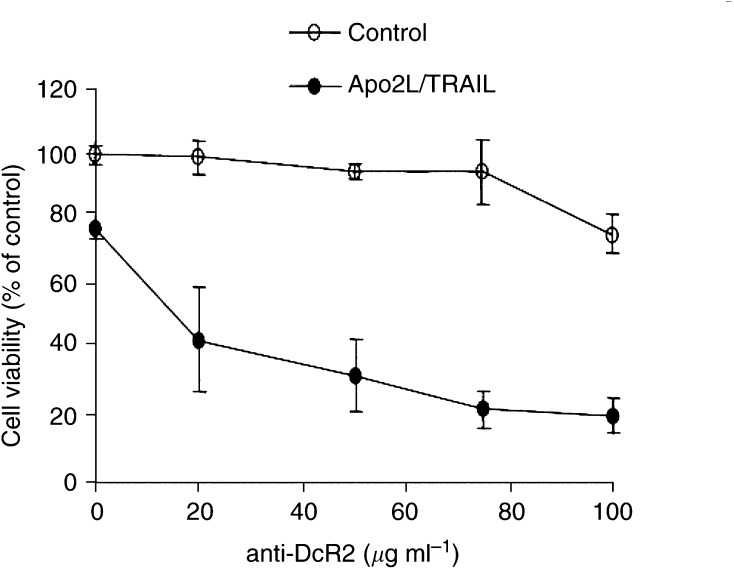
Effect of blocking the function of DcR2 using a specific anti-DcR2 antibody. Cells were incubated with DcR2 antibody alone at increasing concentrations as indicated (open circles) or with anti-DcR2 antibody followed by incubation with 100 ng ml^−1^ recombinant Apo2L/TRAIL (closed circles). Blocking of DcR2 resulted in a dose-dependent resensitisation to Apo2L/TRAIL-induced apoptosis in the late-passage-resistant BTK-143 cells (closed circles) compared with DcR2 antibody treatment alone (open circles). Viability is expressed as a percentage of the viability of untreated control cells. An isotype-matched negative control antibody titrated in a similar manner with Apo2L/TRAIL had no effect (data not shown).

To assess the expression of Apo2L/TRAIL receptors at the protein level, we performed flow cytometric analysis on intact cells using specific antibodies to each of the receptors. Our results clearly demonstrated that cell surface expression of DcR2 increased with passage, and confirmed the data we obtained with RT–PCR analysis (Figure 4A

). There were no detectable changes in the cell surface expression of either of the two death receptors, DR4 and DR5, or the other decoy receptor, DcR1. Immunofluorescence labelling with an antibody directed against DcR2 demonstrated a significantly higher level of DcR2 receptor expression in the late-passaged cells when compared to early-passaged cells. The 1B5 isotype-matched (IgG1) murine monoclonal antibody was used as a negative control (Figure 4B). The expression of DcR2 was predominantly cytoplasmic, with strong expression also at the cell surface. To assess whether the acquired resistance to Apo2L/TRAIL was attributable to the gain in DcR2 expression, blocking antibodies against DcR2 were used in late passage, resistant BTK-143 cells. Preincubation of late-passage cells in the presence of increasing concentrations of the anti-DcR2 monoclonal antibody for a period of 24 h restored in a dose-dependent manner their sensitivity to Apo2L/TRAIL-induced apoptosis (Figure 5

), whereas incubation of the cells with an isotype-matched negative control monoclonal antibody had no effect (data not shown). Taken together, these results suggest that the gain of DcR2 expression confers protection from Apo2L/TRAIL-induced apoptosis in these cells.

### Chemotherapy sensitises resistant BTK-143 cells to Apo2L/TRAIL/-induced apoptosis

**Figure 6:**
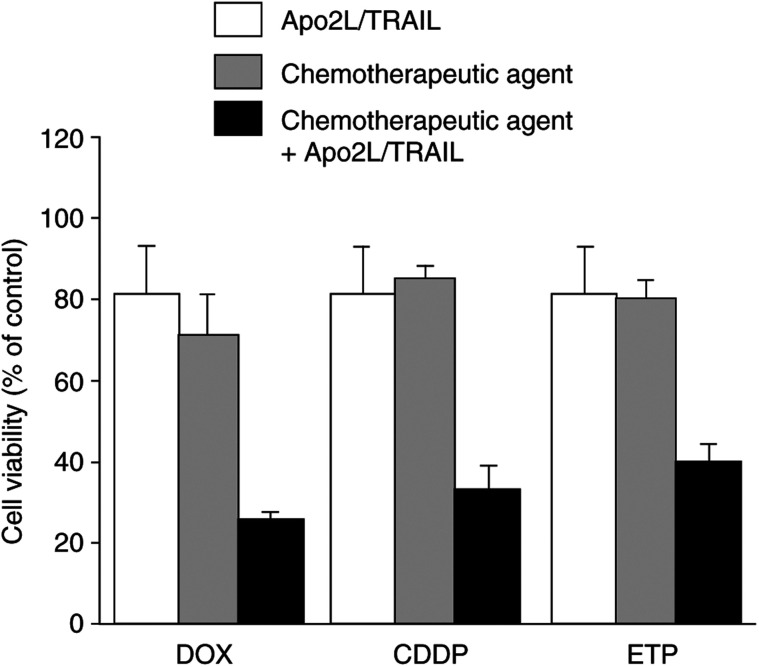
Chemotherapeutic agents DOX, CDDP and ETP sensitised resistant-late passage BTK-143 osteosarcoma cells to Apo2L/TRAIL-induced apoptosis. Passage 19 BTK-143 cells were seeded into multiwell plates, as described for Figure 1, and incubated with Apo2L/TRAIL (100 ng ml) alone, with sublethal doses of chemotherapeutic agent (2.0 *μ*M DOX, 12.5 *μ*M CDDP and 50 *μ*M ETP) alone, or with Apo2L/TRAIL and drug in combination. Cell viability is shown as a percentage of untreated cells after 24 h treatment with Apo2L/TRAIL alone (white bars), drug alone (grey bars), or the combination of drug and Apo2L/TRAIL (black bars). Data points show means of quadruplicate results from a representative experiment, which was repeated three times; bars, ±s.d.

Several reports (Gliniak and Le, 1999; Desjosez *et al*, 2000; Gibson *et al*, 2000; Nagane *et al*, 2000; Yamanaka *et al*, 2000; Lacour *et al*, 2001; Mizutani *et al*, 2001), including our own (Evdokiou *et al*, 2002), have demonstrated that chemotherapeutic drugs augment Apo2L/TRAIL-induced apoptosis of sensitive, and more importantly Apo2L/TRAIL-resistant, cancer cells. Experiments were performed to determine whether combinations of Apo2L/TRAIL with chemotherapeutic agents clinically relevant for the treatment of osteosarcoma, including DOX, CDDP or ETP, could reverse the acquired resistance to Apo2L/TRAIL. The concentration of each agent used in the combined treatment with Apo2L/TRAIL was determined from dose-response curves, and was based on a concentration at which no more than 25% cell death was obtained over a 24 h treatment period (data not shown). The concentrations used in these experiments were as follows: DOX 2.0 *μ*M, CDDP 12.5 *μ*M and ETP 50 *μ*M. Cell viability data (expressed as a percentage of untreated cells), for late-passage (passage 19) BTK-143 cells, incubated with drug alone, or in combination with Apo2L/TRAIL, are shown in Figure 6

**Figure 7:**
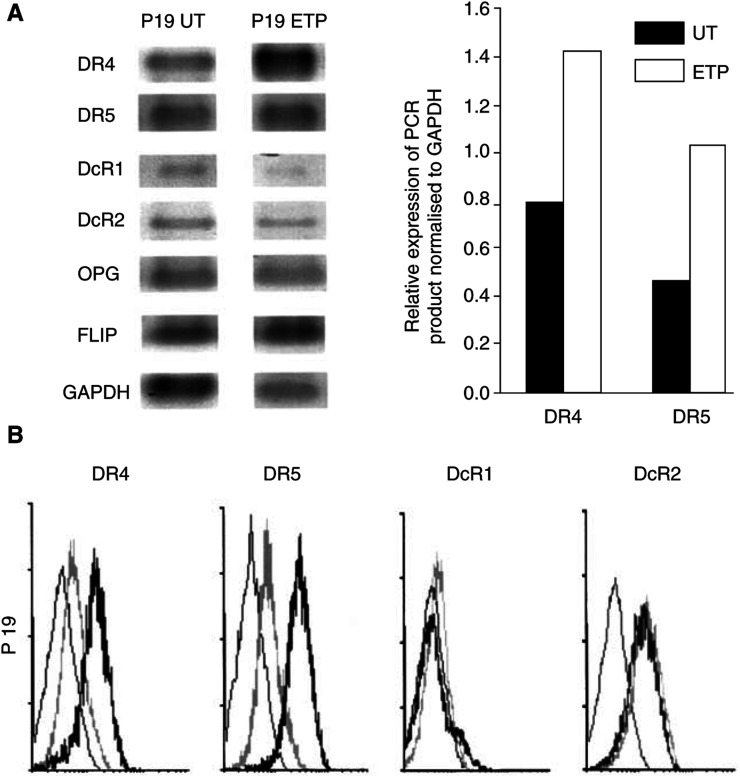
Effect of chemotherapeutic agents on the expression of DR4 and DR5. (**A**) Analysis of Apo2L/TRAIL receptor mRNA expression in the BTK-143 osteosarcoma cells, assessed by semiquantitative RT–PCR analysis, as described in the Materials and Methods. Expression of the housekeeping gene, GAPDH, served to normalise for starting mRNA. Right panel shows the pattern of Apo2L/TRAIL receptor expression for late-passage untreated and ETP-treated BTK-143 cells. Left panel: Fluorimager-generated images of PCR products for DR4 and DR5 shown in the upper panel were quantitated using IMAGEQUANT software (Molecular Dynamics, Sunnyvale, CA, USA). (**B**) Flow cytometric data of cell surface expression of Apo2L/TRAIL receptors shows increased expression of DR4 and DR5 following treatment with ETP (heavy line) compared with untreated cells (grey line). The 1B5 isotype-matched (IgG1) murine monoclonal antibody was used as a negative control (light line).

. While neither Apo2L/TRAIL alone nor chemotherapeutic agent alone had an appreciable effect on late-passage BTK-143 cells, each of the chemotherapeutic agents in combination with Apo2L/TRAIL resulted in a significant increase in cell death, representing a reversal of the resistant state of these cells. Reverse transcriptase–polymerase chain reaction analysis of cells following these treatments did not show any change in the expression of either DcR1 or DcR2 mRNA. However, we found that sensitisation of resistant late passage BTK-143 cells to Apo2L/TRAIL-induced apoptosis by these agents was accompanied by a drug-induced upregulation of both DR4 and DR5 death receptors. Figure 7A

shows an increase in the expression of DR4 and DR5 mRNA upon treatment with ETP over a 24 h period. Similarly, flow cytometric analysis of intact cells, using specific antibodies directed against each of the receptors, clearly demonstrated that expression of DR4 and DR5 was significantly increased following treatment with ETP, whereas expression of DcR1 and DcR2 remained unchanged (Figure 7B). These results confirm the data we obtained with RT-PCR analysis. Resensitisation to Apo2L/TRAIL was also accompanied by an increase in the level of FLIP mRNA (Figure 7A). These data are reminiscent of our previously published work showing that anticancer agents, including ETP, DOX and CDDP, upregulate expression of DR4 and/or DR5 in other osteosarcoma cell lines that are normally resistant to Apo2L/TRAIL and can sensitise these cells to the apoptotic effects of the ligand (Evdokiou *et al*, 2002). In addition, our recent findings in normal human osteoblasts, in which proapoptotic and protective components of the Apo2L/TRAIL machinery appear to be coregulated in response to drugs such as ETP (Atkins *et al*, 2002), suggests that it is the balance of expression of these opposing influences that determines the outcome of exposure to Apo2L/TRAIL. These results show that resistance to Apo2L/TRAIL-induced apoptosis in BTK-143 cells can be overcome with the use of conventional chemotherapeutic agents, and suggest that this may be due at least partly to increased expression of death receptors.

## DISCUSSION

Osteosarcoma accounts for a high percentage of primary malignant tumours of bone. Treatment regimens have been developed in the last decade using cytotoxic chemotherapeutic drugs, which deliver significantly improved prognoses, particularly in younger patients. However, the toxicity of conventional chemotherapy limits its usefulness in older patients. Apo2L/TRAIL has been shown to specifically promote apoptosis in a wide variety of cancer cell types, and has hence been the subject of intense study in recent years.

Apo2L/TRAIL is able to induce apoptosis in a caspase-dependent manner *via* the activation of death receptors (DR4 and DR5). The mechanisms of differential sensitivity to Apo2L/TRAIL of different tumour types, or between tumours of the same type, are not well understood. However, there appear to be multiple mechanisms that apply, including increased expression of the decoy receptors for Apo2L/TRAIL (Griffith *et al*, 1998; Degli-Esposti, 1999; Keane *et al*, 1999; Zhang *et al*, 2000a,2000b), the over-expression of intracellular inhibitory proteins such as FLIP (Griffith *et al*, 1998), intracellular inhibitor of apoptosis molecules (IAPs) (Suliman *et al*, 2001) and the loss of caspase-8 activity by gene methylation (Siegmund *et al*, 2001). Although the inherent expression of the decoy receptors for Apo2L/TRAIL was thought to be the main determinant of Apo2L/TRAIL resistance, it is, however, unlikely to be the sole reason given that we (Evdokiou *et al*, 2002), and others (Degli-Esposti, 1999; Lacour *et al*, 2001), have not been able to demonstrate a consistent correlation between Apo2L/TRAIL receptor expression and sensitivity to Apo2L/TRAIL-induced apoptosis.

It is known that cellular responses to Apo2L/TRAIL depend on a complex interplay between the death and decoy receptors, and possibly OPG, as well as the participation of proapoptotic and prosurvival intracellular molecules such as FADD, FLIP, NFκB and Akt/PKB (Chaudhary *et al*, 1997; Nesterov *et al*, 2001; Ravi *et al*, 2001). It is possible that in a given tissue type, tumour or cell line, it is the balance of the numerous proapoptotic and prosurvival factors that determines the response to Apo2L/TRAIL, and that the perturbation of this balance by a single component may be enough to change the magnitude or the nature of the response.

Our data suggest that the gain in function or upregulation of the decoy receptors, in particular DcR2, may be important in the acquired loss of sensitivity to Apo2L/TRAIL in the osteosarcoma cell line BTK-143. DcR2 expression in BTK-143 cells progressively increased with passage in culture, and this increase correlated with a loss of sensitivity of these cells to Apo2L/TRAIL. Furthermore, blocking the function of DcR2 in the resistant cells resensitised them to Apo2L/TRAIL-induced apoptosis. In light of this result, it is possible that DcR2 is able to provide intracellular antiapoptotic signals, possibly through transcriptional regulation of other antiapoptotic genes, and further experiments will need to be performed to test this possibility.

The reasons for the loss of sensitivity that occurred with continued passaging in the present experiments are unclear, as these cells had presumably been extensively passaged prior to their acquisition for the study reported here. However, the results suggest that the culture conditions used for the present experiments induced DcR2 expression and thus selected for a more resistant phenotype. It is well established that some tumour types, as well as subpopulations of cells within a tumour type, are resistant to Apo2L/TRAIL-induced apoptosis (Bin *et al*, 2002). Also, it has been reported that melanoma cells are frequently resistant at the time of surgical excision, but regain sensitivity in cell culture (Nguyen *et al*, 2001). A similar effect has been demonstrated in platelets that have been stored for transfusion, where there is an early increase in the expression of DcR2, but this increase in expression is not enough to protect platelets from lesion, caused by apoptosis and caspase activation of these cells (Plenchette *et al*, 2001). It is therefore important to develop an understanding of the factors that lead to sensitivity or resistance of tumour cells to Apo2L/TRAIL.

We (Evdokiou *et al*, 2002), and others (Gliniak and Le, 1999; Desjosez *et al*, 2000; Gibson *et al*, 2000; Nagane *et al*, 2000; Yamanaka *et al*, 2000; Lacour *et al*, 2001; Mizutani *et al*, 2001), have shown that Apo2L/TRAIL can be successfully combined with currently used chemotherapeutic treatments to sensitise already resistant cancer cells to Apo2L/TRAIL-induced apoptosis. In the present experiments, combining sublethal concentrations of the chemotherapeutic drugs DOX, CDDP and ETP with Apo2L/TRAIL reversed the loss of sensitivity in BTK-143 cells and restored the initial apoptotic effect. The resensitisation to the effects of Apo2L/TRAIL by chemotherapy was associated with drug-induced upregulation of death receptors DR4 and DR5 at the level of mRNA and protein. This further supports the hypothesis that perturbation of the balance between the expression of death and decoy receptors is important in governing sensitivity to Apo2L/TRAIL-induced apoptosis. Presumably, exposure to sublethal concentrations of chemotherapeutic agents such as those described here, altered the balance of these factors in favour of apoptosis by a process involving upregulation of death receptors. In contrast, we recently showed that normal human osteoblasts maintain viability in response to chemotherapeutic drugs and Apo2L/TRAIL combinations, by coregulating the expression of several antiapoptotic factors as the expression of death receptors is induced (Atkins *et al*, 2002). The molecular mechanisms by which chemotherapeutic drugs induce DR4 and DR5 expression are not yet understood and are under intense investigation by this laboratory. Many anticancer drugs are known to activate the tumour suppressor protein p53, which can in turn upregulate expression of death receptors DR4 and DR5 (Gibson *et al*, 2000). However, we and others have previously shown that chemotherapy can also induce expression of DR4 and/or DR5 in cancer cells that are null or mutant for p53 suggesting that p53-dependent and -independent mechanisms may be involved (Evdokiou *et al*, 2002). The BTK-143 osteosarcoma cell line used in this study expresses p53, but whether this protein is wild type or mutant has not been ascertained.

In summary, our results indicate that the acquisition of function or the upregulation of decoy receptors, in particular DcR2, is important in the loss of sensitivity to Apo2L/TRAIL-induced apoptosis. However, using low concentrations of currently used chemotherapeutic agents in combination with Apo2L/TRAIL, the acquired resistance of these cancer cells was reversed. This implies that future combination therapeutic regimens involving recombinant Apo2L/TRAIL and standard chemotherapeutic drugs for the treatment of osteosarcoma and other cancer types, may not only provide a more effective treatment, but would also require lower doses of drugs than those currently used. The use of such treatment regimens has exciting implications for reducing the frequency and the extent of the morbidity experienced by many cancer patients as a result of both their disease and of their treatment.
